# Effects of Personalized Nutrition Education Tailored to Individual Genetic Risk Profiles on Weight Loss in Adults with Obesity: A Randomized Controlled Trial

**DOI:** 10.3390/healthcare14060766

**Published:** 2026-03-18

**Authors:** Sun Hwa Jung, Yoo Kyoung Park

**Affiliations:** 1 Department of Medical Nutrition, Graduate School of East-West Medical Science, Kyung Hee University, Yongin 17104, Republic of Korea; myhofh777@khu.ac.kr; 2 Department of Medical Nutrition, AgeTech-Service Convergence Major, Kyung Hee University, Yongin 17104, Republic of Korea

**Keywords:** diet therapy, genetic counseling, genetic predisposition to disease, nutrigenetics, obesity, personalized nutrition, polymorphism, weight reduction programs

## Abstract

**Highlights:**

**What are the main findings?**

- Personalized nutrition education tailored to individual genetic risk profiles was associated with greater reductions in body weight, body mass index, waist circumference, and body fat, compared to conventional nutrition education in adults with obesity.
- In exploratory subgroup analyses, participants classified as having higher genetic risk for carbohydrate-related weight gain or glucose regulation demonstrated more pronounced behavioral and metabolic improvements when receiving genotype-informed counseling.

**What are the implications of the main findings?**

- Integrating individual genetic risk information into structured nutrition education may enhance perceived personal relevance and support dietary adherence in obesity management.
- Precision nutrition delivered through genotype-informed educational frameworks may serve as a practical behavioral strategy for sustainable lifestyle modification in real-world settings.

**Abstract:**

**Background/Objectives**: Responses to lifestyle interventions vary widely in obesity, and genetic factors may enhance outcomes. This study evaluated whether a 12-week genotype-informed personalized nutrition education (GEN) program improved weight and overall body composition among adults with obesity. **Methods**: Adults with a body mass index ≥ 25 kg/m^2^ were randomized to a genotype-informed personalized nutrition education (GEN) group or a control group receiving standard nutrition education. The GEN group received weekly counseling tailored to nine obesity-related genetic traits. Changes were evaluated using paired *t*-tests and repeated-measures analysis of variance, with significance defined as *p* < 0.05. **Results**: Forty-three participants (GEN: *n* = 19; CON: *n* = 24) were analyzed. After 12 weeks, the GEN group showed significantly greater reductions than the CON group in body weight (−3.35 ± 0.7 vs. –0.91 ± 0.4 kg, *p* = 0.004), BMI (–1.17 ± 0.3 vs. –0.32 ± 0.1 kg/m^2^, *p* = 0.005), and waist circumference (–5.56 ± 0.8 vs. –2.53 ± 0.7 cm, *p* < 0.001). Energy (–415 kcal, *p* = 0.003) and carbohydrate intake (–65 g, *p* = 0.003) also decreased significantly in the GEN group. Exploratory subgroup analyses suggested that participants classified as high genetic risk showed more pronounced improvements when receiving genotype-informed counseling. No serious adverse events were reported. **Conclusions**: The genotype-informed personalized nutrition program was associated with greater improvements in body composition than general nutrition education. Integrating genetic risk information into structured nutrition education may enhance perceived personal relevance and support effective weight management.

## 1. Introduction

According to the World Health Organization (WHO), obesity refers to a state in which excess adipose tissue accumulates to an extent that negatively influences physiological health and disease risk [1]. As of 2022, one in eight adults worldwide is reported to be obese [2], and the prevalence of obesity in Korea is steadily increasing [3]. Obesity is known to be associated with individual lifestyle habits [4] and has emerged as a considerable public health problem that increases the risk of developing metabolic syndrome, hypertension, cardiovascular disease, arthritis, and certain cancers [5]. The causes of the rapid increase in obesity remain controversial; however, environmental and behavioral changes, including diet, physical activity, and the built environment, have been identified as major contributing factors [6,7]. To address this issue, various treatments, including diet, exercise, medication, and surgery, have been developed and implemented in real-life settings for decades. However, conventional interventions often apply a standardized approach, which frequently fails to maintain long-term weight loss. Notably, in many intervention studies, some individuals have even reported regaining more weight than initially lost [5,8]. These results suggest substantial variation in responses between individuals.

A landmark study published in 2015 demonstrated that individuals exhibit markedly different glycemic responses to the same foods [9]. These findings highlight the limitations of standardized strategies and underscore the need for personalized approaches that account for individual differences in metabolism, genetics, and lifestyle. Recent evidence indicates that incorporating genetic information into personalized nutritional interventions for individuals with obesity may improve dietary adherence, enhance nutrient intake following dietary modifications, and ultimately promote better health outcomes [10,11,12]. Among the many genes associated with obesity risk, the fat mass and obesity-associated (FTO) gene has been consistently identified as one of the most considerable contributors [13]. Although the relationship among FTO variants, dietary influences, and weight gain remains unclear, certain variants have been suggested to influence the risk of obesity by increasing food intake or altering appetite and satiety regulation [14,15]. This biological variability necessitates a shift from generalized public health messages to targeted nutritional strategies.

These insights are supported by a growing body of research regarding gene–diet interactions and the emerging field of nutrigenetics. The primary goal of nutrigenetics is to refine standard nutritional recommendations by accounting for genetic variations, thereby enabling a more individualized level of dietary guidance [16,17,18]. In addition to FTO, >250 genetic loci related to body mass index (BMI) and obesity have been identified. Furthermore, studies utilizing direct-to-consumer genetic testing have provided encouraging evidence for its potential role in supporting weight loss and maintenance [19]. Accordingly, efforts are underway to integrate genetic information into personalized healthcare and nutritional interventions.

Lifestyle interventions, comprising dietary modification along with greater levels of physical activity, continue to form the basis of obesity management. Although their efficacy is well-established, a key challenge in clinical practice is the marked variability between individuals in treatment response. In routine settings, some individuals achieve clinically meaningful weight loss, whereas others experience only modest changes or rapid weight regain despite following identical protocols. This heterogeneity is regarded as a major contributor to poor long-term adherence and high attrition rates in conventional obesity programs [20,21,22].

Emerging evidence has explored whether genetic variation may partly account for differences in individual responses to dietary interventions. Variants related to appetite regulation, macronutrient metabolism, and glucose homeostasis may influence how individuals respond to specific dietary prescriptions [23,24,25]. Against this backdrop, nutrigenetics has gained attention as an effective approach to align biological individuality with lifestyle modification. Adapting dietary advice according to an individual’s genetic profile has been shown to increase the perceived personal relevance of recommendations, thereby enhancing motivation and treatment adherence [11,26]. Large initiatives such as the Food4Me, POINTS, and MyGeneMyDiet trials provide preliminary support for this concept, indicating that genetics-informed nutritional counseling can lead to greater improvements in eating behaviors and weight control than standard, non-personalized guidance [11,12,27].

Nevertheless, translating these findings into routine clinical practice remains challenging. Results from randomized controlled trials are not yet fully concordant, and important gaps persist in the evidence base. Many prior studies have relied on broad genetic risk scores, exclusively web-based interventions, or short follow-up periods, which may limit their generalizability to real-world community settings [19,28]. In particular, research on structured genotype-informed personalized nutrition education remains limited, especially in Asian populations, and robust clinical evidence in Korean adults with obesity is therefore still lacking [29].

Therefore, this study aimed to evaluate, through a 12-week randomized controlled trial, whether personalized nutritional counseling based on genetic risk and dietary data is more effective than standard information provision for weight reduction among Korean adults with obesity. Rather than validating a polygenic risk prediction model, the focus was on how genotype information was applied within a structured personalized nutrition counseling program. We hypothesized that participants receiving genotype-informed personalized nutrition education (GEN group) would exhibit greater improvements in overall body composition (e.g., body weight and BMI) and selected metabolic biomarkers compared with those receiving standard nutrition education (CON group), with more pronounced effects among participants classified as higher genetic risk who received genotype-informed counseling. Beyond a simple dietary intervention, this study sought to provide empirical evidence on how lifestyle interventions that incorporate genotype-informed educational content contribute to obesity management in community settings.

## 2. Materials and Methods

### 2.1. Study Design and Participants

This study was conducted over 3 months from February to May 2021. Eligible participants were adults aged ≥30 years who voluntarily consented to participate after receiving information about the study purpose. Recruitment was carried out through public health centers in the Seoul metropolitan area, bulletin boards at local community centers, and an online medical community (the Hi-Doc platform). Participants were included as having obesity according to the Korean and Asian-Pacific criteria (BMI ≥ 25 kg/m^2^), which are commonly applied in Asian populations due to higher metabolic risk at lower BMI thresholds compared with Western populations.

Participants were excluded if they had any of the following conditions: (1) a history of diabetes, stroke, malignant tumors, cardiovascular disease, hypertension, psychiatric disorders, or current use of medications for these conditions; (2) recent use of weight-control diets, dietary supplements, or medications related to weight management within the preceding 3 months; (3) scheduled overseas travel within the next 3 months; (4) pregnancy or plans for pregnancy; and (5) engagement in regular physical activity for >1 h per day. Participants who experienced discomfort from continuous glucose monitoring, developed health or residential issues, or voluntarily withdrew during the study period were classified as dropouts.

**Figure 1:**
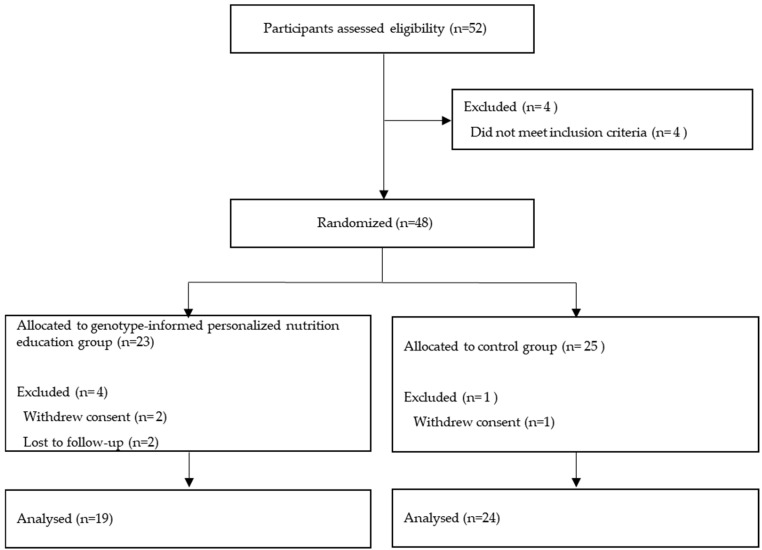
Study Flow Diagram.

The sample size was calculated to detect a clinically meaningful decrease in body weight of 2.5 kg (standard deviation [SD] = 3.5 kg, effect size dz = 0.71) from diet-only interventions. This estimation was derived from findings reported in previous studies examining weight reduction outcomes in comparable personalized nutrition interventions [22,30]. In particular, the effect size from Kwon et al. [22] was conservatively adjusted for the present analysis, who reported a weight reduction of −6.2 kg with diet plus exercise. Using G*Power software (version 3.1) for a paired *t*-test (α = 0.05, power = 80%), 17 participants were required. Considering the 20% attrition rate observed in nutritional intervention studies without exercise, we recruited 22 participants per group. The sample size calculation was intended to detect statistically significant differences in the primary outcome (weight loss) and was not powered to evaluate post hoc subgroup analyses. A total of 52 individuals applied to participate, and four did not meet the eligibility criteria, leaving 48 participants enrolled. After identification numbers were assigned in order of registration, participants were randomly allocated to either the genotype-informed personalized nutrition education group (GEN) or the control group (CON). The random allocation sequence was generated using computer-generated random numbers by an independent researcher with no involvement in participant recruitment and was kept confidential until all participants were fully enrolled. Given the characteristics of the nutritional counseling intervention, blinding of the intervention could not be implemented. In contrast, assessors conducting anthropometric measurements and laboratory analyses were masked to group assignment. During the intervention period, five participants withdrew due to scheduling difficulties or health-related issues unrelated to the intervention. Therefore, data from 43 participants (GEN, *n* = 19; CON, *n* = 24) were retained for the final analysis (Figure 1).

The study was performed in compliance with the ethical standards of the Declaration of Helsinki and received approval from the Institutional Review Board of Kyung Hee University on 19 January 2021 (IRB No. KHGIRB-21-029). It was registered in the Clinical Research Information Service (CRIS registration number: KCT0011415) on 6 January 2026.

### 2.2. Intervention

**Figure 2:**
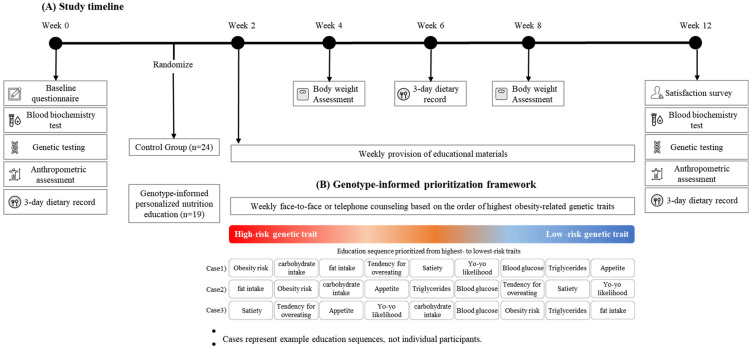
Study timeline and conceptual framework of genotype-informed prioritization used in the GEN group. (**A**) Study timeline illustrating assessment points and intervention delivery over the 12-week period. (**B**) Conceptual framework showing how nutrition education in the GEN group was prioritized according to individual genetic risk profiles. Participants were classified into high-, moderate-, or low-risk categories for multiple obesity-related genetic traits. Nutrition education was then sequenced from the highest-risk trait to the lowest-risk trait rather than delivered in a fixed order. Cases 1–3 are illustrative examples demonstrating how the same educational components were reordered differently depending on the dominant genetic risk. These cases are schematic and do not represent individual participants.

The intervention lasted 12 weeks (Figure 2). Participants in the control group (CON) received general nutrition education materials based on standard obesity management guidelines, delivered once weekly via non-face-to-face methods. In contrast, participants in the GEN group received personalized nutritional counseling and educational materials tailored to their obesity-related genetic risk traits. Of the 12 sessions, four were conducted as face-to-face, one-on-one counseling sessions at the institution, while the remaining sessions involved weekly delivery of educational materials based on obesity-related genetic traits. In addition, participants in the GEN group received a 20 min telephone consultation once per week to ensure continuous nutrition management.

In this study, genotype information was used solely to prioritize and sequence nutritional education content according to individual genetic risk profiles, rather than to categorize participants into distinct groups or apply group-specific interventions.

### 2.3. Measurements

#### 2.3.1. Study Protocol

A randomized controlled trial with a 12-week intervention period was conducted, with identical assessments conducted at baseline (week 0) and at the end of the intervention (week 12). After a minimum overnight fast of 7 h, venous blood samples were collected to measure fasting glucose, hemoglobin A1c (HbA1c), insulin, alanine aminotransferase (ALT), aspartate aminotransferase (AST), C-reactive protein (CRP), total cholesterol, low-density lipoprotein cholesterol (LDL-C), high-density lipoprotein cholesterol (HDL-C), and triglycerides (TG). Anthropometric measurements included body weight, skeletal muscle mass, fat mass, percent body fat, BMI, waist circumference, hip circumference, and waist–hip ratio (WHR). Participants also completed a baseline questionnaire and a 3-day dietary record.

#### 2.3.2. Nutrition Intervention and Exercise Monitoring

(1) Nutrition Education and Counseling

All participants received weekly nutrition education materials. Participants in the CON group received only general obesity management guidelines, whereas those in the GEN group received additional individualized counseling. Counseling sessions for the GEN group lasted approximately 20 min per week, with four sessions conducted in person and the remaining sessions conducted by telephone.

Participants assigned to the GEN group documented their dietary intake using a 3-day dietary record and a questionnaire before the start of the study to assess eating habits and tendencies toward overeating or binge eating. Based on this information, individualized nutrition plans were developed by considering each participant’s standard body weight and activity level. To promote an approximate weekly weight loss of 0.5 kg, daily energy intake was prescribed to be reduced by 500 kcal. 

Counseling sessions were conducted using educational materials developed according to the study protocol, including general obesity management guidelines and genetically tailored dietary recommendations based on individual obesity-related genetic traits (Table 1). A detailed comparison of the educational structure and delivery between the CON and GEN groups is provided in Supplementary Table S1. During each session, counseling incorporated participants’ genetic analysis results, blood and body composition measurements, dietary habits, and analysis of the 3-day food log. Participants reviewed the educational materials and provided feedback at each visit. In addition, nutritional analysis results from the most recent 3-day dietary record were compared with previous results to monitor changes over time.

(2) Exercise Monitoring

All participants received a weekly 20 min exercise video created step-by-step by a professional trainer to support self-guided practice. For the GEN group, adherence to the exercise program was assessed at each nutrition counseling session.

(3) Anthropometric and Muscle Strength Measurements

Body weight was measured four times: at baseline (week 0), and weeks 4, 8, and 12. Body composition, waist circumference, hip circumference, and WHR were assessed twice, at baseline and at the end of the intervention (week 12). For all anthropometric assessments, participants wore light clothing and no shoes. Height was measured using a stadiometer while the participant stood upright and was recorded to the nearest 0.1 cm. Body weight was measured using the same device and recorded to the nearest 0.1 kg. In the CON group, participants self-reported body weight at weeks 4 and 8. Interim body weight at these time points was self-measured by participants using personal scales and submitted with photographic verification. Participants received information regarding the clinical purpose of the study and were instructed to ensure accurate reporting of all measurements. BMI (kg/m^2^) was calculated by dividing body weight (kg) by height squared (m^2^).

Additionally, grip strength was measured 2 times for each right and left hand using a dynamometer (Jamar, Plus + Digital Hand Dynamometer, Preferred, New York City, NY, USA), and the average value was recorded.

(4) Blood Analysis

Blood sampling was conducted at two time points: baseline (week 0) and week 12,after a minimum fasting period of 8 h. At each visit, 9 mL of blood was drawn, and all laboratory analyses were performed by a certified commercial laboratory, LabGenomics (Seongnam, Republic of Korea).

Total cholesterol, HDL-C, TG, AST, ALT, and fasting glucose levels were measured using enzymatic assays. CRP levels were assessed by immunoturbidimetry, insulin levels by enzyme-linked immunosorbent assay, and HbA1c levels by high-performance liquid chromatography. LDL-C was calculated based on the Friedewald equation: total cholesterol—HDL-C—(TG/5). Any remaining samples were discarded immediately after analysis [31].

(5) DNA Test

A direct-to-consumer DNA test was conducted once at baseline (week 0) using a blood sample. Nine obesity-related genetic markers were analyzed, including obesity risk, carbohydrate metabolism, fat intake, overeating tendency, satiety, appetite regulation, the yo-yo effect, blood sugar control, and triglyceride metabolism. For each marker, results were classified into three categories: high-, moderate-, and low-risk. All genetic analyses were performed using LabGenomics software (Seongnam, Republic of Korea). The genetic panel was structured to reflect commercially available direct-to-consumer genetic reports and was not intended to serve as a validated polygenic risk prediction model.

(6) Dietary Intake

Dietary intake, including total energy, protein, fat, fiber, vitamins, and minerals, was assessed using a 3-day food record. These assessments were conducted three times: at baseline (week 0), at mid-intervention (week 6), and at the end of the study (week 12). At baseline, a trained dietitian provided instructions on completing the food records, and participants documented all foods consumed and the eating location across two weekdays and one weekend. For the GEN group, additional details, such as ingredients and cooking methods, were reviewed individually during one-on-one counseling sessions. All dietary records were analyzed using a web-based, computer-assisted nutrient analysis program (CAN-Pro version 5.0; Korean Nutrition Society, Seoul, Republic of Korea).

**Table 1:** Nutrition Education Content Tailored to Genetic Trait Profiles.

Genetic Trait	Gene Marker	Gene Description	Gene Marker Targeted Education Contents
Obesity risk	FTO, CLOCK	Variants in FTO and CLOCK are associated with energy metabolism, circadian rhythm, and an increased risk of obesity [20,21].	Education focused on balancing calorie intake and expenditure, reducing carbohydrate intake, and improving sleep quality to manage fat accumulation.
Weight gain from carbohydrate intake	PLIN(Perilipin 1)	PLIN1 polymorphisms, which regulate lipid droplet metabolism, interact with dietary carbohydrate and glycemic load, influencing insulin sensitivity and body fat accumulation [24].	Education included the impact of carbohydrate intake on fat storage and insulin response, with emphasis on reducing sugars and refined carbohydrates.
Weight gain from fat intake	APOA2, APOA5, APOA4, FTO	Variants in APOA2, APOA5, and FTO interact with dietary fat intake and are linked to higher BMI and triglyceride levels [23,25,26].	Participants were advised to limit intake of saturated and trans fats and were provided with practical strategies for healthier fat choices.
Tendency for overeating	ANKK1	ANKK1 is associated with dopamine receptors and affects reward-based eating behaviors. Variants are linked to increased susceptibility to overeating and the consumption of addictive foods [28,32].	Education focused on portion control and managing stress-induced overeating through behavioral strategies.
Satiety	FTO	FTO influences hormones secreted by the brain and gastrointestinal tract that regulate satiety, leading to greater energy intake before the sensation of fullness is achieved [33].	Guidance included slowing down eating, choosing fiber- and protein-rich foods, and adopting eating behaviors that enhance satiety.
Appetite	TAS2R38, ANKK1	TAS2R38 is associated with taste sensitivity, which affects food preferences and dietary choices [34]. ANKK1 variants also regulate appetite through the dopamine signaling pathway [28,32].	Education included drinking water before meals, controlling portion sizes, and brushing teeth after meals to help reduce appetite.
Yo-yo effect likelihood	FBLN5, LAMB1, POSTN	Genetic variants in FBLN5, LAMB1, and POSTN have been linked to an increased likelihood of weight regain following weight loss, suggesting an underlying genetic contribution susceptibility to the yo-yo effect [35].	Education emphasized avoiding extreme diets and maintaining long-term weight through exercise, dietary consistency, and adequate sleep.
Blood glucose	DGKB, GCK, SLC30A8, CDKN2A/B, MTNR1B	These genes play key roles in the regulation of glucose metabolism, insulin secretion, and pancreatic β-cell function. Genetic variants within these loci have been linked to higher fasting glucose levels, impaired glucose tolerance, and an elevated risk of type 2 diabetes [36].	Education involved reading food labels for carbohydrate content, minimizing high- glycemic index (GI) foods, and selecting fiber-rich options to manage postprandial glucose.
Triglycerides	ANGPTL3, GCKR, TBL2, MLXIPL, TRIB1	These genes are involved in triglyceride synthesis, lipid metabolism, and hepatic fat regulation. Risk variants have been linked to hypertriglyceridemia and dyslipidemia in clinical studies [37,38].	Participants were guided to reduce sugar and alcohol intake and adopt fat-controlled diets tailored to individual needs.

### 2.4. Statistical Analysis

All data were analyzed using IBM SPSS Statistics for Windows (version 23.0; IBM Corp., Armonk, NY, USA). Baseline characteristics are presented as means ± standard deviation (SD), whereas changes over time are expressed as means ± standard errors. Categorical variables are presented as frequencies and percentages. Changes from baseline to post- and mid-intervention were assessed using paired *t*-tests. When assumptions of normality were not met, Wilcoxon signed-rank tests were performed. Differences between categorical variables were examined using chi-square tests, and differences in continuous variables over time and between groups were analyzed using repeated-measures analysis of variance. Subgroup analyses based on genetic risk categories (e.g., high-risk) were conducted as post hoc exploratory analyses. Due to the limited sample size in these subgroups, these analyses were intended to generate hypotheses rather than confirm efficacy. Differences between subgroups were evaluated using independent t-tests. All statistical tests were two-tailed, and a *p*-value < 0.05 was considered statistically significant.

## 3. Results

### 3.1. General Characteristics of the Participants

This study was conducted from February to May 2021. A total of 43 participants (GEN: *n* = 19; CON: *n* = 24) were included in the final analysis. Five participants withdrew during the study period. In the GEN group, four participants (17.4%) withdrew due to personal logistical reasons, specifically residential relocation and difficulties in coordinating face-to-face counseling sessions with work schedules. In the CON group, one participant (4.0%) withdrew. Fisher’s exact test indicated that the difference in dropout rates between the two groups was not statistically significant (*p* > 0.05). Baseline characteristics of the study participants are summarized in Table 2. The overall mean age was 41.9 years, with mean ages of 42.40 ± 11.48 years in the GEN group and 41.60 ± 10.85 years in the CON group, with no significant difference between the two groups (*p* = 0.735). Overall, 41.9% of participants were female, with no significant difference in sex distribution (*p* = 0.977). Most participants had previously attempted weight loss, reported by 84.2% of the GEN group and 83.3% of the CON group, with no significant difference between groups (*p* = 0.635). No significant differences were observed between groups in age, height, body weight, smoking status, alcohol consumption, or exercise habits.

### 3.2. Changes in Body Composition, Anthropometric Measurements, and Blood Biomarkers

**Figure 3:**
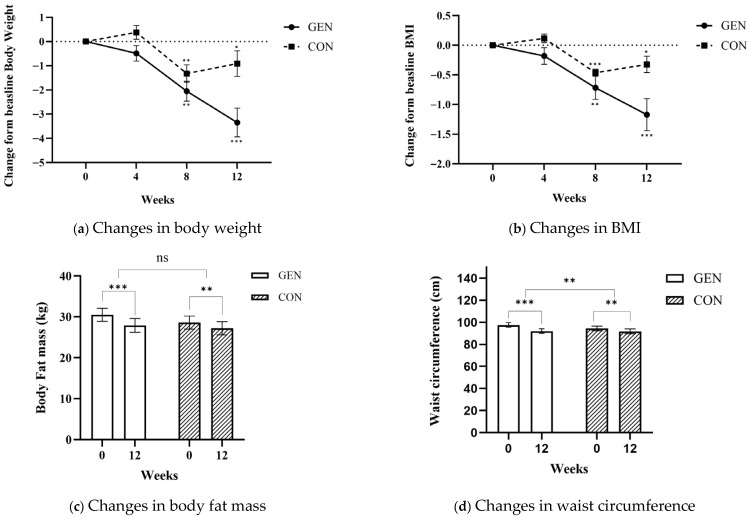
Changes in anthropometric and body composition parameters over the 12-week intervention. (**a**) Body weight; (**b**) BMI; (**c**) Body fat mass; (**d**) Waist circumference. Values are presented as mean ± standard error (SE). Changes from baseline were calculated at weeks 4, 8, and 12 (Δ4–0, Δ8–0, Δ12–0). Within-group changes from baseline were assessed using paired t-tests, and between-group differences in change were assessed using independent *t*-tests. * *p* < 0.05, ** *p* < 0.01, *** *p* < 0.001. Abbreviations: BMI, body mass index; GEN, genotype-informed personalized nutrition education group; CON, control group.

As shown in Figure 3a–d, both the GEN and CON groups demonstrated significant improvements in body composition after the 12-week intervention.

Body weight decreased significantly in both groups; however, the reduction was greater in the GEN (−3.35 ± 0.70 kg) than in the CON group (−0.91 ± 0.40 kg, *p* < 0.001). Similarly, BMI decreased by −1.17 ± 0.30 kg/m^2^ in the GEN and −0.32 ± 0.10 kg/m^2^ in the CON group, with significant between-group differences emerging after week 8 (*p* < 0.01). Body fat mass also decreased significantly within both groups (GEN: −2.64 ± 0.50 kg, *p* < 0.001; CON: −1.39 ± 0.40 kg, *p* < 0.001), though the between-group difference approached but did not reach statistical significance (*p* = 0.051). Waist circumference showed a pronounced reduction in the GEN (−5.56 ± 0.80 cm, *p* < 0.001) compared with the CON group (−2.53 ± 0.70 cm, *p* = 0.001), with a significant between-group difference (*p* = 0.007). Although both groups showed significant improvements from baseline, reductions in body weight, BMI, waist circumference, and body fat mass were more pronounced in the GEN group. Over time, the GEN group showed significant decreases in body weight and BMI by week 4, which were maintained through week 12. In contrast, the CON group showed only modest improvements, with a slight increase observed after week 8.

Detailed numerical values for anthropometric and body composition outcomes are presented in Supplementary Table S2, and changes in blood biomarkers are summarized in Table S3. HbA1c levels decreased significantly in both groups (GEN: −0.15 ± 0.17%; CON: −0.18 ± 0.23%; *p* = 0.001). Comparisons between the GEN and CON groups revealed no significant differences in fasting glucose, insulin, CRP, ALT, AST, or lipid measures, including total cholesterol, TG, LDL-C, and HDL-C.

In addition, a descriptive analysis was conducted to examine transitions from abnormal to normal reference ranges for selected biochemical markers (Supplementary Table S4). Among participants with elevated HbA1c at baseline, more individuals in the GEN group transitioned to the normal range compared with the CON group.

### 3.3. Subgroup Analyses According to Genetic Risk

As an exploratory post hoc analysis, subgroup analyses were conducted to examine whether participants classified as high-risk for selected genetic traits showed different response patterns to the intervention. Based on the nine direct-to-consumer genetic markers used in this study, two traits—weight gain related to carbohydrate intake and glucose regulation—were selected for exploratory subgroup analyses. These analyses were descriptive in nature and were not intended to provide confirmatory evidence. Because Table 3 and Table 4 present baseline characteristics of participants classified as high-risk for specific genetic traits, baseline values in these tables may differ from those shown for the full randomized sample in Table 2.

**Table 3:** Changes in Body Composition, Blood Biomarkers, and Nutrient Intake According to Genetic Traits Related to Weight Gain from Carbohydrate Intake after the 12-Week Intervention.

		GEN (*n* = 10)				CON (*n* = 14)				*p-*Value Δ12 w − 0 w
		0 Week	12 Week	Δ12 w − 0 w	*p-*Value	0 Week	12 Week	Δ12 w − 0 w	*p-*Value	
Body Composition										
	Body weight (kg)	85.60 ± 16.80	80.80 ± 16.70	−4.81 ± 1.47	0.005 **	79.40 ± 17.70	78.70 ± 18.00	−0.67 ± 1.89	0.208	0.001 ***
	BMI (kg/m^2^)	30.00 ± 3.30	28.30 ± 3.30	−1.72 ± 0.58	0.003 **	28.90 ± 3.60	28.70 ± 3.90	−0.24 ± 0.68	0.207	0.001 ***
	Skeletal muscle mass (kg)	31.20 ± 8.40	30.50 ± 8.00	−0.76 ± 0.64	0.001 ***	28.00 ± 7.30	28.40 ± 7.80	0.41 ± 0.74	0.058	0.001 **
	Body fat mass (kg)	30.10 ± 8.20	26.60 ± 8.10	−3.49 ± 1.71	0.001 ***	29.30 ± 8.20	27.90 ± 8.40	−1.33 ± 2.04	0.030 *	0.012 *
	Percent body fat (%)	35.40 ± 8.50	33.00 ± 8.40	−2.35 ± 1.89	0.107	36.90 ± 5.80	35.60 ± 6.30	−1.29 ± 1.75	0.016 *	0.173
	Waist circumference (cm)	99.00 ± 8.70	92.00 ± 9.70	−7.02 ± 2.54	0.001 ***	93.90 ± 11.00	90.60 ± 11.40	−2.94 ± 3.28	0.002 *	0.003 **
	Hip circumference (cm)	107.60 ± 5.80	102.90 ± 5.50	−4.71 ± 1.75	0.003 **	104.50 ± 6.90	101.60 ± 8.30	−2.49 ± 2.09	0.001 ***	0.012 *
	WHR	0.90 ± 0.00	0.90 ± 0.10	−0.03 ± 0.02	0.005 **	0.90 ± 0.10	0.90 ± 0.10	−0.01 ± 0.03	0.404	0.065
Nutrient Intake										
	Absolute intake									
	Energy (Kcal)	1844.60 ± 547.80	1237.40 ± 174.6	−607.24 ± 519.23	0.003 **	1911.10 ± 572.20	1805.30 ± 700.20	−105.84 ± 554.52	0.488	0.035 *
	Carbohydrate (g)	236.20 ± 62.40	149.00 ± 42.7	−87.28 ± 67.07	0.001 **	216.90 ± 52.50	189.50 ± 53.70	−27.35 ± 75.88	0.200	0.058
	Protein (g)	69.80 ± 26.00	66.00 ± 18.2	−3.75 ± 24.68	0.882	82.10 ± 27.60	76.20 ± 28.00	−5.95 ± 40.36	0.590	0.880
	Fat (g)	64.40 ± 35.10	40.70 ± 10.6	−23.72 ± 33.1	0.053	60.10 ± 16.80	58.40 ± 18.30	−1.66 ± 17.22	0.724	0.077
	Fiber (g)	14.90 ± 4.50	14.60 ± 4.6	−0.36 ± 4.44	0.802	19.10 ± 6.20	15.70 ± 3.90	−3.38 ± 6.87	0.088	0.237
	Macronutrient distribution (% of total energy)									
	Carbohydrate (%)	54.00 ± 10.90	36.00 ± 15.40	−17.99 ± 11.85	0.046 *	50.00 ± 6.80	45.10 ± 15.00	−4.93 ± 16.84	0.293	0.047 *
	Carbohydrate intake classification (relative to recommended range)									
	Carbohydrate ratio	3.60 ± 1.10	2.50 ± 1.40	−1.09 ± 1.49	0.050	2.80 ± 0.80	2.60 ± 0.80	−0.18 ± 1.15	0.566	0.105
Blood Biomarkers										
	Glucose (mg/dL)	91.60 ± 11.40	93.10 ± 10.50	1.50 ± 7.01	0.038 *	87.90 ± 14.50	95.00 ± 13.00	7.07 ± 8.97	0.011 *	0.116
	HbA1c (%)	5.60 ± 0.30	5.50 ± 0.20	−0.15 ± 0.20	0.655	5.70 ± 0.60	5.40 ± 0.60	−0.28 ± 0.19	0.001 ***	0.124
	Insulin (μIU/mL)	12.80 ± 9.20	11.10 ± 8.30	−1.71 ± 7.41	0.585	12.80 ± 5.40	10.20 ± 5.50	−2.56 ± 5.68	0.116	0.752
	HOMA-IR	1.10 ± 0.20	1.10 ± 0.10	−0.02 ± 0.10	0.309	1.10 ± 0.30	1.10 ± 0.30	0.03 ± 0.13	0.441	0.399
	CRP (mg/dL)	0.20 ± 0.20	0.20 ± 0.10	−0.06 ± 0.08	0.516	0.20 ± 0.20	0.20 ± 0.10	−0.02 ± 0.17	0.640	0.511
	ALT (IU/L)	36.20 ± 28.50	28.60 ± 17.50	−7.60 ± 14.49	0.798	33.10 ± 16.20	30.10 ± 17.90	−2.93 ± 14.32	0.458	0.441
	AST (IU/L)	25.80 ± 9.50	25.20 ± 8.30	−0.60 ± 7.18	0.784	27.90 ± 8.70	27.80 ± 12.60	−0.14 ± 9.11	0.954	0.896
	Total cholesterol (mg/dL)	219.50 ± 40.50	217.00 ± 28.90	−2.50 ± 28.02	0.051	211.70 ± 30.90	210.00 ± 31.00	−1.71 ± 24.47	0.979	0.942
	Triglyceride (mg/dL)	157.90 ± 87.70	142.10 ± 68.80	−15.80 ± 88.3	0.001 ***	208.60 ± 106.00	267.50 ± 333.20	58.86 ± 294.18	0.467	0.447
	LDL cholesterol (mg/dL)	134.90 ± 36.40	133.50 ± 24.10	−1.44 ± 28.54	0.485	118.60 ± 26.90	104.70 ± 62.20	−13.84 ± 52.05	0.338	0.503
	HDL cholesterol (mg/dL)	53.00 ± 12.10	55.10 ± 11.20	2.10 ± 6.15	0.877	51.40 ± 8.00	51.80 ± 11.20	0.36 ± 9.25	0.887	0.610

Carbohydrate intake classification was defined as follows: 1 = intake below the recommended range (55–65% of total energy); 2 = intake within the recommended range; 3 = intake above the recommended range. Values are presented as means ± standard deviation (SD). * *p* < 0.05, ** *p* < 0.01, *** *p* < 0.001. BMI, body mass index; WHR, waist-to-hip ratio; HbA1c, hemoglobin A1c; HOMA-IR, homeostatic model assessment for insulin resistance; GEN, genotype-informed personalized nutrition education; CON, control group; CRP, C-reactive protein; ALT, alanine aminotransferase; AST, aspartate aminotransferase; LDL-C, low-density lipoprotein cholesterol; HDL-C, high-density lipoprotein cholesterol; TG, triglycerides; Kcal, kilocalories. The *p*-value (12 w–0 w) represents the between-group comparison of changes from baseline to week 12 (GEN vs. CON).

**Table 4:** Changes in Body Composition, Blood Biomarkers, and Nutrient Intake According to Genetic Trait Related to Weight Gain from Glucose after the 12-Week Intervention.

		GEN (*n* = 7)				CON (*n* = 4)				*p-*Value Δ12 w − 0 w
		0 Week	12 Week	Δ12 w − 0 w	*p-*Value	0 Week	12 Week	Δ12 w − 0 w	*p-*Value	
Body Composition										
	Body weight (kg)	87.90 ± 15.60	84.00 ± 14.50	−3.91 ± 2.24	0.004 **	97.70 ± 20.60	97.00 ± 21.20	−0.73 ± 2.96	0.658	0.073
	BMI (kg/m^2^)	30.70 ± 2.90	29.30 ± 2.80	−1.34 ± 0.75	0.003 **	31.70 ± 5.20	31.50 ± 5.50	−0.23 ± 0.92	0.652	0.056
	Skeletal muscle mass (kg)	29.90 ± 7.20	29.40 ± 6.70	−0.51 ± 0.71	0.105	36.50 ± 2.90	36.70 ± 2.80	0.28 ± 0.61	0.432	0.097
	Body fat mass (kg)	34.40 ± 5.70	31.30 ± 5.20	−3.03 ± 1.21	0.001 **	33.80 ± 15.80	32.60 ± 16.40	−1.20 ± 2.38	0.387	0.119
	Percent body fat (%)	39.40 ± 4.90	37.60 ± 4.70	−1.77 ± 0.66	0.001 ***	33.20 ± 8.90	32.10 ± 9.30	−1.10 ± 1.3	0.190	0.392
	Waist circumference (cm)	101.10 ± 11.50	94.80 ± 11.20	−6.29 ± 3.05	0.002 **	103.50 ± 13.50	101.50 ± 14.20	−2.03 ± 1.08	0.033 *	0.027 *
	Hip circumference (cm)	107.90 ± 6.50	104.00 ± 6.00	−3.90 ± 2.65	0.008 **	110.40 ± 10.10	107.10 ± 12.60	−3.25 ± 2.90	0.111	0.713
	WHR	0.90 ± 0.10	0.90 ± 0.10	−0.03 ± 0.03	0.071	0.90 ± 0.00	0.90 ± 0.00	0.01 ± 0.02	0.322	0.066
Nutrient Intake										
	Absolute intake									
	Energy (Kcal)	1687.40 ± 347.90	1268.00 ± 198.90	−419.38 ± 263.9	0.006 **	2369.10 ± 755.70	2180.70 ± 573.00	−188.45 ± 785.82	0.664	0.604
	Carbohydrate (g)	221.00 ± 44.30	142.30 ± 40.60	−78.70 ± 43.32	0.003 **	212.50 ± 54.50	192.40 ± 37.40	−20.06 ± 68.91	0.601	0.113
	Protein (g)	64.00 ± 15.80	59.90 ± 14.40	−4.08 ± 14.74	0.492	118.60 ± 42.20	91.80 ± 37.10	−26.83 ± 26.25	0.134	0.093
	Fat (g)	45.20 ± 13.40	44.00 ± 14.20	−1.18 ± 19.68	0.879	84.40 ± 27.20	80.50 ± 48.10	−3.91 ± 27.75	0.796	0.852
	Fiber (g)	12.20 ± 3.50	11.70 ± 3.70	−0.55 ± 4.99	0.781	19.40 ± 5.90	25.80 ± 20.80	6.47 ± 17.7	0.518	0.49
	Macronutrient distribution (% of total energy)									
	Carbohydrate (%)	57.30 ± 5.40	37.10 ± 9.20	−20.22 ± 11.23	0.003 **	41.00 ± 3.70	39.20 ± 13.00	−1.86 ± 14.29	0.812	0.042 *
	Carbohydrate intake classification (relative to recommended range)									
	Carbohydrate ratio	3.50 ± 0.60	2.50 ± 0.90	−1.02 ± 0.97	0.032 *	1.80 ± 0.30	2.30 ± 0.90	0.45 ± 0.60	0.231	0.024 *
Blood Biomarkers										
	Glucose (mg/dL)	95.00 ± 9.50	93.60 ± 7.7	−1.43 ± 14.03	0.797	85.80 ± 9.40	95.80 ± 3.70	10.00 ± 9.83	0.135	0.188
	HbA1c (%)	5.60 ± 0.20	5.40 ± 0.10	−0.19 ± 0.13	0.011 *	5.50 ± 0.30	5.40 ± 0.30	−0.10 ± 0.14	0.252	0.344
	Insulin (μIU/mL)	12.10 ± 4.60	11.00 ± 6.50	−1.14 ± 5.85	0.625	16.00 ± 6.70	12.20 ± 3.70	−3.76 ± 4.3	0.179	0.457
	HOMA-IR	1.20 ± 0.10	1.10 ± 0.10	−0.06 ± 0.16	0.384	1.00 ± 0.10	1.10 ± 0.10	0.10 ± 0.12	0.200	0.122
	CRP (mg/dL)	0.10 ± 0.00	0.30 ± 0.40	0.16 ± 0.42	0.36	0.10 ± 0.10	0.20 ± 0.10	0.05 ± 0.06	0.182	0.631
	ALT (IU/L)	34.70 ± 20.20	31.70 ± 18.80	−3.00 ± 3.51	0.065	39.00 ± 18.10	44.80 ± 27.20	5.75 ± 10.56	0.356	0.196
	AST (IU/L)	24.10 ± 5.60	26.40 ± 9.50	2.29 ± 5.94	0.348	29.50 ± 12.00	36.30 ± 22.70	6.75 ± 13.00	0.375	0.446
	Total cholesterol (mg/dL)	219.10 ± 39	213.00 ± 31.70	−6.14 ± 28.47	0.589	199.80 ± 25.90	195.50 ± 33.20	−4.25 ± 9.22	0.424	0.876
	Triglyceride (mg/dL)	126.10 ± 69.50	149.10 ± 66.80	23.00 ± 69.32	0.414	230.80 ± 149.00	246.50 ± 189.70	15.75 ± 48.71	0.564	0.859
	LDL cholesterol (mg/dL)	135.30 ± 27.60	127.30 ± 24.90	−8.03 ± 20.57	0.342	104.90 ± 11.80	97.50 ± 13.00	−7.40 ± 8.08	0.164	0.955
	HDL cholesterol (mg/dL)	58.60 ± 15.10	55.90 ± 16.00	−2.71 ± 3.25	0.069	48.80 ± 4.80	48.80 ± 2.90	0.00 ± 2.31	1.000	0.179

Carbohydrate intake classification was defined as follows: 1 = intake below the recommended range (55–65% of total energy); 2 = intake within the recommended range; 3 = intake above the recommended range. Values are presented as means ± standard deviation (SD). * *p* < 0.05, ** *p* < 0.01, *** *p* < 0.001. BMI, body mass index; WHR, waist-to-hip ratio; HbA1c, hemoglobin A1c; HOMA-IR, homeostatic model assessment for insulin resistance; GEN, genotype-informed personalized nutrition education group; CON, control group; CRP, C-reactive protein; ALT, alanine aminotransferase; AST, aspartate aminotransferase; LDL-C, low-density lipoprotein cholesterol; HDL-C, high-density lipoprotein cholesterol; TG, triglycerides; Kcal, kilocalories. The *p*-value (12 w − 0 w) represents the between-group comparison of changes from baseline to week 12 (GEN vs. CON).

#### 3.3.1. Weight Gain from Carbohydrate Intake

As presented in Table 3, this exploratory post hoc analysis examined participants classified as high-risk for obesity-related genetic traits associated with weight gain from carbohydrate intake. In both the GEN and CON groups, numerical reductions in body weight and BMI were observed over the 12-week intervention period. Larger reductions were observed in the GEN group (−4.81 ± 1.47 kg and −1.72 ± 0.58 kg/m^2^, respectively) compared with the CON group (−0.67 ± 0.89 kg and −0.24 ± 0.68 kg/m^2^; *p* < 0.001). Waist and hip circumferences showed a similar directional pattern, with larger numerical reductions in the GEN group (waist: −7.02 ± 2.54 cm and hip: −4.71 ± 1.75 cm) compared with the CON group (waist: −2.94 ± 3.28 cm and hip: −2.49 ± 2.09 cm). Given the limited sample size in this subgroup, these findings are presented descriptively and without confirmatory inference.

Regarding nutrient intake, the GEN group showed numerical reductions in total energy intake (−607.24 ± 519.23 kcal) and carbohydrate intake (−87.30 ± 67.10 g; *p* < 0.01), along with a decrease in the proportion of carbohydrates in the diet (−18.00 ± 11.90%). Smaller reductions were observed in the CON group.

Regarding blood biomarkers, the GEN group showed modest numerical decreases in HbA1c, insulin, and indices of insulin resistance assessed using the homeostatic model, although these changes did not reach statistical significance. A directional reduction in triglyceride levels was observed in the GEN group (−15.80 ± 88.30 mg/dL; *p* < 0.001); however, given the exploratory nature of this analysis, this finding should be interpreted with caution.

#### 3.3.2. Weight Gain Related to Glucose Regulation

As presented in Table 4, this exploratory post hoc analysis examined participants classified as high-risk for obesity-related genetic traits associated with glucose regulation. In the GEN group, numerical reductions were observed in body weight (−3.91 ± 2.42 kg, *p* = 0.004) and BMI (−1.34 ± 0.75 kg/m^2^, *p* = 0.003) over the 12-week intervention period. Reductions were also observed in the CON group (−0.73 ± 2.96 kg and −0.23 ± 0.92 kg/m^2^), although these changes did not reach statistical significance. Waist circumference showed a similar pattern: the GEN group experienced a significant decrease (−6.02 ± 3.56 cm; *p* = 0.002), whereas the CON group showed a modest reduction (−2.94 ± 3.08 cm; *p* = 0.033). Given the very small sample size in this subgroup (GEN: *n* = 7; CON: *n* = 4), these findings are presented descriptively without confirmatory inference.

Regarding dietary intake, the GEN group showed numerical reductions in total energy (−419.30 ± 382.60 kcal; *p* = 0.006) and carbohydrate intake (−64.80 ± 43.30 g; *p* = 0.003). The CON group also showed decreases, but these were smaller and did not reach statistical significance (energy: −188.40 ± 575.80 kcal; carbohydrate: −20.10 ± 66.90 g). No consistent directional changes were observed for other macronutrients, including protein, fat, and fiber.

For blood biomarkers, a directional decrease in HbA1c was observed in the GEN group (−0.13 ± 0.13%; *p* = 0.011), whereas no clear change was observed in the CON group. Changes in insulin levels were modest and did not reach statistical significance in either group.

## 4. Discussion

This randomized controlled trial is among the early studies conducted in Korea to evaluate how a genotype-informed personalized nutrition education affects body composition in adults with obesity. Over the 12-week intervention, participants in the GEN group demonstrated greater improvements in body weight as well as body composition than the control group, consistent with the primary outcomes reported in the Results section. This suggests that integrating genotype information into counseling may contribute to enhanced behavioral engagement and adherence within the context of this intervention. Furthermore, this approach appears to be more effective than general education alone in driving dietary adjustments.

These findings indicate that nutritional counseling based on genetic information can provide more than basic education. It may support behavioral adjustments and the adoption of healthier eating habits, rather than serving solely as educational guidance. The outcomes of this study are also consistent with earlier research showing that personalized nutrition interventions improve body composition more effectively than general nutrition education [39]. A similar pattern was observed in the Food4Me trial, involving seven European countries; in that study, online personalized nutrition advice improved eating behaviors and supported weight reduction more effectively than standard guidance [27]. Other trials, such as POINTS and MyGeneMyDiet, also showed that programs using genetic information improved dietary adherence and facilitated weight loss [11,12]. Taken together, these studies support our findings and suggest that a genotype-informed personalized nutrition education approach may contribute to meaningful behavioral and metabolic improvements.

Our findings suggest that the effectiveness of the intervention was driven not merely by the provision of genetic data, but by how this information was integrated into the educational process. While conventional obesity management often relies on broad, generalized recommendations, the present intervention structured counseling according to individual genetic risk classifications to guide prioritization. This targeted structure allowed key behavioral goals to be addressed repeatedly and with greater relevance, helping participants translate general dietary advice into concrete, personally meaningful actions. In this context, genetic information did not function as an independent determinant of weight loss, but rather as a framework that shaped the sequencing and emphasis of nutrition education. Such in-depth, tailored guidance may enhance engagement with dietary recommendations, perceived relevance, and self-efficacy, which have been identified as important drivers of sustained behavioral change in genetics-informed lifestyle interventions [11,37].

Although previous studies have explored genetics-based interventions, many have relied on broad disease risk markers or nutrient metabolism variants, with limited specificity for obesity-related outcomes [40]. Conversely, the present study implemented a targeted strategy focusing on genetic traits directly linked to metabolic regulation and eating behaviors. By tailoring nutritional counseling to these specific profiles, the intervention sought to address individual vulnerabilities more effectively, suggesting the potential clinical utility of this structured, genotype-informed educational model.

In subgroup analyses, body composition showed favorable changes in both groups; however, participants classified as higher genetic risk who received genotype-informed counseling demonstrated clearer behavioral and metabolic changes. For example, among participants carrying the high-risk PLIN1 variant associated with weight gain from carbohydrate intake, genotype-informed personalized nutrition education emphasizing carbohydrate reduction was accompanied by larger decreases in carbohydrate intake and greater improvements in body weight and body fat mass. These observations are consistent with previous gene–diet interaction studies reporting that PLIN1, which encodes perilipin, plays a key role in lipid droplet metabolism and interacts with carbohydrate and fat intake to influence insulin sensitivity and adiposity [41,42]. However, given the exploratory nature and limited sample size of the present subgroup analysis, these findings should be interpreted with caution. The rs894160 variant has also been reported to interact with carbohydrate and fat intake to influence insulin sensitivity, waist circumference, and overall fat accumulation [42], supporting the gene–diet interaction observed in this study. Participants classified as higher genetic risk for glucose regulation (DGKB, GCK, SLC30A8, CDKN2A/B, and MTNR1B) tended to exhibit more favorable glycemic patterns when receiving genotype-informed counseling, suggesting that tailored feedback may support blood glucose management in this subgroup. These observations are consistent with reports that these genes contribute to glucose metabolism and the progression to type 2 diabetes [43,44]. Overall, the subgroup results suggest that genotype-informed counseling may be particularly helpful for individuals classified as having higher metabolic risk related to obesity, although these findings remain exploratory.

These findings suggest that individuals classified as higher genetic risk within this study framework may experience meaningful behavioral changes and metabolic improvements when receiving genotype-informed counseling. Genetic information can function not only as a predictive marker but also as a motivating factor that encourages healthier choices, aligning with the core principles of precision nutrition.

These results also indicate that precise nutritional approaches to weight and glycemic management may be more effective when genetic information is integrated with phenotypic characteristics [9,45]. This underscores the need for personalized approaches that integrate genetic, metabolic, and behavioral data.

While most biochemical markers remained within normal ranges, descriptive analyses suggested a greater normalization of HbA1c in the GEN group, reflecting early metabolic changes rather than definitive clinical improvement.

Lifestyle factors play a crucial role in shaping nutritional requirements and health outcomes [46]. Measures such as anthropometry and dietary intake provide important information about overall health and can guide the development of personalized dietary plans informed by individual genetic characteristics [47]. Popova [11] found that providing genetic feedback enhanced individuals’ perceived control and motivation for lifestyle modification, resulting in stronger behavioral responses. In this context, the metabolic differences observed in this study suggest that genetic feedback may extend beyond identifying disease risk, catalyzing behavioral change and measurable metabolic improvements.

Taken together, these findings suggest that genotype-informed personalized nutrition education may serve as a practical behavioral intervention tool that goes beyond providing information, helping individuals understand their genetic vulnerabilities and adopt sustainable lifestyle changes in daily health management contexts.

From an applied perspective, the findings of this study suggest that genotype-informed personalized nutrition education may be feasibly integrated into real-world obesity management programs. In particular, this approach may be well suited for use in community health centers, primary care clinics, and commercial wellness services that already provide direct-to-consumer genetic testing. By using genetic risk information to prioritize and contextualize nutrition education, practitioners may enhance participant engagement and adherence without requiring complex clinical genomics infrastructure. This model supports the practical implementation of precision nutrition as an educational and behavioral intervention rather than as a diagnostic tool, thereby expanding its applicability in routine lifestyle counseling settings.

This study has several limitations. First, the relatively small sample size and unequal group allocation may limit the generalizability of the findings. While the study was adequately powered for detecting the primary outcome (weight loss), the sample size was not sufficient to support post hoc subgroup analyses. As a result, gene-specific findings based on high-risk subgroups should be interpreted cautiously and considered exploratory and hypothesis-generating in nature.

Second, because the intervention integrated genotype-informed educational content delivered through individualized counseling and follow-up consultations, the independent effect of genetic information cannot be fully separated from the effects of increased contact time and personalized support. Accordingly, the observed benefits should be interpreted as the combined effect of genotype-informed education and its delivery format rather than the isolated impact of genetic information alone.

Third, the absence of follow-up after the intervention precludes a robust assessment of the long-term sustainability of the observed effects. In addition, although a few biochemical measures showed statistically significant changes, most remained within normal physiological ranges, limiting their clinical relevance.

Future studies with larger sample sizes, balanced intervention intensity, and longer follow-up periods are needed to better clarify the independent and sustained effects of genotype-informed personalized nutrition interventions. It is also important for future research to move beyond simple gene categories and adopt a more comprehensive precision-nutrition approach that includes genetic, metabolic, and behavioral factors.

## 5. Conclusions

This study is the first randomized controlled trial in Korea to evaluate genotype-informed personalized nutrition education in adults with obesity. The findings showed that counseling informed by obesity-related genetic traits, delivered through structured weekly sessions, was associated with greater improvements than general nutritional education for improving body composition. Participants in the GEN group experienced greater improvements across key anthropometric measures, including body weight, body fat percentage, and waist circumference, compared with the CON group.

Exploratory subgroup analyses suggested that responses varied among participants classified according to genetic risk categories. Among those classified as higher genetic risk for weight gain from carbohydrate intake, participants receiving genotype-informed counseling demonstrated greater reductions in carbohydrate intake as well as in body weight and body fat. Similarly, participants classified as higher genetic risk for glucose regulation showed improvements in HbA1c when receiving genotype-informed counseling. These findings suggest that tailoring nutritional strategies based on genotype information may enhance participant engagement and improve the effectiveness of obesity management, although these results should be interpreted cautiously.

These findings also highlight the practical value of genotype-informed personalized nutrition education beyond a research setting. When genetic feedback is incorporated into structured counseling, individuals may perceive dietary advice as more personally relevant, which could enhance motivation for behavior change. This approach can be incorporated into health management tools that integrate genetic, dietary, and lifestyle information, thereby providing a foundation for applying precision nutrition in real-world settings.

Future studies should include a wider range of genetic markers and examine whether behavioral and metabolic improvements are sustained over time. Such research is important for establishing the practical use of precision nutrition in obesity management. 

## Figures and Tables

**Table 2:** Baseline Characteristics of the Study Participants.

	Total (*n* = 43)	GEN (*n* = 19)	CON (*n* = 24)	*p-*Value
Sex				
Male	25 (58.10)	11 (57.90)	14 (58.30)	0.977
Female	18 (41.90)	8 (42.10)	10 (41.70)	
Age (year)	41.90 ± 11.00	42.40 ± 11.48	41.60 ± 10.85	0.735
Smoking				
Current smoker	8 (18.60)	2 (10.50)	6 (25.00)	0.479
Past smoker	6 (14.00)	3 (15.80)	3 (12.50)	
None	29 (67.40)	14 (73.70)	15 (62.50)	
Drinking				
Yes	32 (74.40)	14 (73.70)	18 (75.0)	0.922
No	11 (25.60)	5 (26.30)	6 (25.00)	
Weight control experience				
Yes	36 (83.70)	16 (84.20)	20 (83.30)	0.635
No	7 (16.30)	3 (15.80)	4 (16.70)	
Exercise				
Yes	17 (39.50)	5 (26.30)	12 (50.00)	0.103
No	26 (60.50)	14 (73.70)	12 (50.00)	
Anthropometric measures				
Height (cm)	168.20 ± 9.30	168.20 ± 9.21	168.30 ± 9.57	0.477
Body weight (kg)	83.40 ± 15.61	84.50 ± 13.90	82.50 ± 17.08	0.344
BMI (kg/m^2^)	29.20 ± 3.20	29.70 ± 2.98	28.90 ± 3.39	0.428
Skeletal muscle mass (kg)	30.20 ± 7.22	30.30 ± 6.92	30.20 ± 7.60	0.386
Body fat mass (kg)	29.50 ± 7.44	30.50 ± 7.05	28.60 ± 7.77	0.382
Percent body fat (%)	35.50 ± 6.74	36.30 ± 7.25	34.90 ± 6.39	0.427
Waist circumference (cm)	95.90 ± 9.78	97.60 ± 9.34	94.50 ± 10.09	0.595
Hip circumference (cm)	105.90 ± 5.91	106.70 ± 5.39	105.30 ± 6.33	0.475
WHR	0.90 ± 0.06	0.90 ± 0.07	0.90 ± 0.06	0.428

GEN, genotype-informed personalized nutrition education group; CON, control group; significantly different at *p* < 0.05 by independent *t*-test, chi-square test (Fisher’s exact test); values are N (%); values are mean ± standard deviation (SD); BMI, Body Mass Index; WHR, Waist-to-Hip Ratio.

## Data Availability

The data supporting the findings of this study are available from the corresponding author upon reasonable request. Due to privacy and ethical considerations concerning personal health and genetic information, only de-identified data can be shared.

## Supplementary Materials

The following supporting information can be downloaded at: https://www.mdpi.com/article/10.3390/healthcare14060766/s1. Supplementary Table S1 presents a detailed comparison of educational curricula between the control and genetic groups. Supplementary Table S2 provides detailed changes in anthropometric measurements and body composition following the 12-week intervention. Supplementary Table S3 summarizes changes in blood biomarkers over 12 weeks. Supplementary Table S4 reports changes in the number of metabolic risk factors among participants.

## Abbreviations

The following abbreviations are used in this manuscript:

ALT	alanine aminotransferase
AST	aspartate aminotransferase
BMI	body mass index
CON	control group
CRP	C-reactive protein
GEN	genotype-informed personalized nutrition education group
GI	glycemic index
HbA1c	hemoglobin A1c
HDL-C	high-density lipoprotein cholesterol
HOMA-IR	homeostatic model assessment of insulin resistance
LDL-C	low-density lipoprotein cholesterol
SD	standard deviation
TG	triglycerides
WHR	waist-to-hip ratio
