# Supplementary material for: Effects of Personalized Nutrition Education Tailored to Individual Genetic Risk Profiles on Weight Loss in Adults with Obesity: A Randomized Controlled Trial
Source: Healthcare (Basel). 2026 Mar 18;14(6):766. doi: 10.3390/healthcare14060766 (PMC13026531; doi:10.3390/healthcare14060766)
Supplement: Supplementary file 1 [file healthcare-14-00766-s001.zip › healthcare-4099585-supplementary.pdf]

Tables S1. Detailed Comparison of Educational Curricula between Control and Genetic Groups.

**Category**	**Control Group (CON)**	**Genetic-trait-based Group (GEN)**
Primary approach	Standardized nutrition education	Genotype-informed personalized nutrition education
Educational framework	General obesity management guidelines	Same core guidelines with genetic risk–based prioritization
Delivery format	Standardized educational materials	Individualized 1:1 counseling (face-to-face and telephone)
Use of genetic information	Not applied	Used to rank obesity-related genetic risks and guide counseling order
Curriculum structure	Fixed curriculum delivered in the same order to all participants	Flexible curriculum; topics reordered and emphasized according to each participant's highest genetic risk
Educational content	Core topics applied uniformly to all participants	Same core topics selectively emphasized based on genetic risk profile
Examples of emphasis	General advice on balanced diet and lifestyle	Early and intensive focus on carbohydrate quality, glucose-related eating behaviors, or appetite control in participants with corresponding high-risk traits
Counseling objective	General lifestyle modification	Targeted modification of behaviors related to highest-risk genetic traits

Core Educational Topics Provided to Both Groups

1. Goal setting and Calorie requirements
2. Balanced diet guide
3. Eating order for glucose control (Veg-Protein-Carb)
4. Risks of alcohol consumption
5. Tips for dining out
6. Lifestyle management (Sleep, Stress)
7. How to read nutrition labels
8. Healthy snacking and Sugar reduction
9. Good vs. Bad Carbohydrates
10. Sodium intake and obesity
11. Healthy cooking methods
12. Weight maintenance Q&A

Tables S2. Changes in Anthropometric Measurements and Body Composition after the 12-Week Intervention

	GEN (n=19)				CON (n=24)				*p-value Δ12w-0w*
	0 week	12 weeks	Δ12w-0w	*p-value*	0 week	12 weeks	Δ12w-0w	*p-value*[3)]	
Body weight (kg)	84.50±13.90	81.20±13.10	*-3.35±3.23*	*0.001****	82.50±17.10	81.50±17.30	*-0.91±1.93*	*0.030**	*0.004***
BMI (kg/m^2)	29.70±3.00	28.50±2.80	*-1.17±1.18*	*0.001****	28.90±3.40	28.50±3.60	*-0.32±0.67*	*0.028**	*0.005***
Skeletal muscle mass (kg)	30.30±6.90	29.90±6.50	*-0.40±0.92*	*0.074*	30.20±7.60	30.50±7.80	*0.30±0.79*	*0.073*	*0.010**
Body fat mass (kg)	30.51±7.10	27.90±7.20	*-2.64±2.32*	*0.001****	28.60±7.80	27.20±7.70	*-1.39±1.75*	*0.001***	*0.051*
Percent body fat (%)	36.30±7.30	34.40±7.60	*-1.85±1.87*	*0.001****	34.90±6.40	33.60±6.00	*-1.28±1.46*	*0.001****	*0.264*
Waist circumference (cm)	97.60±9.30	92.10±9.10	*-5.56±3.59*	*0.001****	94.50±10.10	91.80±11.10	*-2.53±3.46*	*0.001***	*0.007***
Hip circumference (cm)	106.70±5.40	103.40±5.60	*-3.34±2.67*	*0.001****	105.30±6.30	102.50±7.30	*-2.59±2.16*	*0.001****	*0.313*
WHR	0.90±0.10	0.90±0.10	*-0.02±0.03*	*0.001***	0.90±0.10	0.90±0.10	*0.00±0.03*	*0.731*	*0.012**
Right hand grip (kg)	31.60±11.80	32.60±12.10	*0.97±3.47*	*0.241*	35.60±12.60	34.50±13.20	*-1.12±4.38*	*0.222*	*0.097*
Left hand grip (kg)	30.50±12.00	31.10±12.20	*0.68±5.06*	*0.568*	33.10±11.70	33.20±12.60	*0.05±4.22*	*0.950*	*0.662*

Values are presented as mean ± standard deviation (SD). *p < 0.05, **p < 0.01, ***p < 0.001. BMI, body mass index; WHR, waist-to-hip ratio; GEN, genotype-informed personalized nutrition education; CON, control group.

Tables S3. Changes in Blood Biomarkers over 12 Weeks

	GEN (n=19)				CON (n=24)				*p-value Δ12w-0w*
	0 week	12 weeks	Δ12w-0w	*p-value*	0 week	12 weeks	Δ12w-0w	*p-value*	
Glucose (mg/dL)	91.70±10.90	92.70±9.30	*0.95±11.24*	*0.718*	90.20±11.80	91.90±11.20	*1.80±10.62*	*0.428*	*0.812*
HbA1c (%)	5.60±0.30	5.40±0.20	*-0.15±0.17*	*0.001***	5.50±0.50	5.10±0.50	*-0.18±0.23*	*0.001***	*0.619*
Insulin (uIU/mL)	13.20±8.00	11.60±8.10	*-1.60±5.61*	*0.229*	12.40±5.10	9.70±4.90	*-2.65±4.50*	*0.008***	*0.500*
HOMA-IR	1.10±0.20	1.10±0.10	*-0.02±0.14*	*0.559*	1.10±0.30	1.10±0.20	*-0.01±0.13*	*0.605*	*0.907*
CRP (mg/dL)	0.20±0.10	0.20±0.30	*0.03±0.27*	*0.615*	0.20±0.20	0.20±0.40	*0.03±0.21*	*0.564*	*0.928*
ALT (IU/L)	38.70±23.20	32.90±18.20	*-5.84±12.79*	*0.062*	32.80±14.70	28.30±15.10	*-4.42±12.27*	*0.091*	*0.712*
AST[1] (IU/L)	27.20±8.70	27.00±8.70	*-0.26±6.76*	*0.867*	25.90±7.60	25.80±10.40	*-0.13±7.04*	*0.931*	*0.948*
Total cholesterol (mg/dL)	223.30±37.10	217.10±35.60	*-6.26±26.37*	*0.314*	211.70±34.90	208.80±33.50	*-2.88±20.96*	*0.508*	*0.641*
Triglyceride (mg/dL)	155.20±75.50	145.51±68.30	*-9.68±74.09*	*0.576*	199.20±96.10	235.20±261.50	*36.00±227.24*	*0.446*	*0.406*
LDL cholesterol (mg/dL)	138.30±30.50	133.00±26.80	*-5.33±24.59*	*0.358*	120.90±29.90	111.30±49.50	*-9.66±42.34*	*0.275*	*0.694*
HDL cholesterol (mg/dL)	54.00±11.80	55.00±11.80	*1.00±5.91*	*0.470*	50.90±9.80	50.50±9.80	*-0.42±7.87*	*0.798*	*0.518*

Values are presented as mean ± standard deviation (SD). *p < 0.05, **p < 0.01, ***p < 0.001. GEN, genotype-informed personalized nutrition education; CON, control group; HbA1c, hemoglobin A1c; HOMA-IR, homeostatic model assessment for insulin resistance; CRP, C-reactive protein; ALT, alanine aminotransferase; AST, aspartate aminotransferase; LDL-C, low-density lipoprotein cholesterol; HDL-C, high-density lipoprotein cholesterol; TG, triglycerides.

Tables S4. Changes in the number of metabolic risk factors of participants

**Biomarker**	**Group**	**Participants with risk factors at baseline (n)**	**Participants with risk factors after 12 weeks (n)**	**Percent of patients free of risk factor (%)**
HbA1c (≥ 5.7%)	CON	6	**6**	**0.00%**
	**GEN**	**6**	**1**	**83.30%**
Fasting Glucose (≥ 100 mg/dL)	CON	2	1	50.00%
	**GEN**	**5**	**2**	**60.00%**
Total Cholesterol (≥ 200 mg/dL)	CON	15	11	26.70%
	GEN	13	11	15.40%
LDL Cholesterol (≥ 130 mg/dL)	CON	5	3	40.00%
	GEN	12	10	16.70%
Triglycerides (≥ 150 mg/dL)	CON	14	10	28.60%
	GEN	9	7	22.20%

Values are presented as descriptive counts and percentages. No statistical comparisons were performed due to small subgroup sizes.
